# Risk factors for neonatal hypoxic ischemic encephalopathy and therapeutic hypothermia: a matched case-control study

**DOI:** 10.1186/s12884-024-06596-8

**Published:** 2024-06-12

**Authors:** Suoma Roto, Irmeli Nupponen, Ilkka Kalliala, Marja Kaijomaa

**Affiliations:** 1 Department of obstetrics and gynecology, Helsinki University Women’s Hospital, Haartmaninkatu 2, Helsinki, 00029 Finland; 2 Children’s Hospital, University of Helsinki and Helsinki University Hospital, Helsinki, Finland

**Keywords:** Birth asphyxia, Delivery, Hypoxic-ischemic encephalopathy, Induction of labour, Oxytocin treatment, Shoulder dystocia, Therapeutic hypothermia

## Abstract

**Background:**

Peripartum asphyxia is one of the main causes of neonatal morbidity and mortality. In moderate and severe cases of asphyxia, a condition called hypoxic-ischemic encephalopathy (HIE) and associated permanent neurological morbidities may follow. Due to the multifactorial etiology of asphyxia, it may be difficult prevent, but in term neonates, therapeutic cooling can be used to prevent or reduce permanent brain damage. The aim of this study was to assess the significance of different antenatal and delivery related risk factors for moderate and severe HIE and the need for therapeutic hypothermia.

**Methods:**

We conducted a retrospective matched case-control study in Helsinki University area hospitals during 2013–2017. Newborn singletons with moderate or severe HIE and the need for therapeutic hypothermia were included. They were identified from the hospital database using ICD-codes P91.00, P91.01 and P91.02. For every newborn with the need for therapeutic hypothermia the consecutive term singleton newborn matched by gender, fetal presentation, delivery hospital, and the mode of delivery was selected as a control. Odds ratios (OR) between obstetric and delivery risk factors and the development of HIE were calculated.

**Results:**

Eighty-eight cases with matched controls met the inclusion criteria during the study period. Maternal and infant characteristics among cases and controls were similar, but smoking was more common among cases (aOR 1.46, CI 1.14–1.64, *p* = 0.003). The incidence of preeclampsia, diabetes and intrauterine growth restriction in groups was equal. Induction of labour (aOR 3.08, CI 1.18–8.05, *p* = 0.02) and obstetric emergencies (aOR 3.51, CI 1.28–9.60, *p* = 0.015) were more common in the case group. No difference was detected in the duration of the second stage of labour or the delivery analgesia.

**Conclusions:**

Smoking, induction of labour and any obstetric emergency, especially shoulder dystocia, increase the risk for HIE and need for therapeutic hypothermia. The decisions upon induction of labour need to be carefully weighed, since maternal smoking and obstetric emergencies can hardly be controlled by the clinician.

**Supplementary Information:**

The online version contains supplementary material available at 10.1186/s12884-024-06596-8.

## Background

Peripartum asphyxia, generally referred to as birth asphyxia, is one of the main causes of neonatal mortality worldwide [1]. Approximately three to five newborns per 1000 live births in developed countries are affected by birth asphyxia [2]. This condition of hypoxia and acidemia can develop gradually during pregnancy and lead to an emergency cesarean section when detected. It can also develop abruptly when complications during labour occur [3].

The pathophysiology of birth asphyxia and its multifactorial antecedents are well studied and recognized: An increased risk is associated with maternal health problems such as diabetes mellitus, cholestasis of pregnancy, anemia, and hypertension, as well as fetal conditions like intrauterine growth restriction and infections [4, 5]. Extensive effort is made to screen and follow-up these mothers and pregnancies with known obstetric risk factors for development of birth asphyxia.

The clinical signs associated with birth asphyxia may be transient and reversible or lead to permanent neurological impairment or death [6]. A condition called hypoxic-ischemic encephalopathy may follow and, if diagnosed, can further be divided in mild, moderate, and severe [7]. A quick recovery, normal level of consciousness, mild neurological signs and absence of seizures are typical to a mild HIE, whereas moderate and severe HIE include presence of seizures, multiorgan failure, primitive reflexes and altered level of consciousness and tone [7]. The diagnosis of severe birth asphyxia is set when the neonate presents with a five-minute Apgar score of 0 to 3 and a pH of 7.0 or less in the umbilical artery blood sample [4].

In the severe cases of birth asphyxia, HIE predisposes the resuscitated neonate to permanent neurologic morbidities such as cerebral palsy, epilepsy, and developmental delays. The medical intervention to reduce brain damage in term neonates with moderate and severe HIE is therapeutic hypothermia, i.e., cooling of the neonates to around 33 °C for three days [8].

Despite the high-quality maternal care and the recognition of antenatal risks, birth asphyxia and HIE remain a challenge in perinatal care. Due to the multifactorial nature of fetal distress [9, 10], the adverse outcome is not always predictable in risk pregnancies. In addition, many cases of HIE occur unanticipated in low-risk pregnancies.

The aim of this study was to assess the importance of different obstetric risk factors associated with moderate and severe HIE and the need for therapeutic hypothermia in term neonates delivered at the hospitals of Helsinki University Hospital area. We particularly focused on the management protocols of pregnancy and delivery.

## Methods

This was a retrospective, matched case-control study concerning pregnancies and deliveries in the Helsinki University Hospital area. The same guidelines for follow-up and treatment of pregnancy and delivery are used in all Helsinki University area hospitals. The neonatal intensive care is centralized at the Neonatal Intensive Care Unit (NICU) in Helsinki University Hospital Women’s Clinic. The study period was from January 1, 2013, to December 31, 2017. The treatment of deliveries in the Helsinki University Hospital area was re-organized after a closure of one delivery hospital in late 2017 and the patient record systems was changed in early 2020. Due to the possible bias caused by these factors, years after 2017 were excluded from the study.

The study group consisted of patients who gave birth to asphyxiated singleton neonates with aforementioned symptoms of moderate or severe HIE. Each neonate was born term (one case of 36 6/7 gestation weeks), was admitted to the NICU and offered therapeutic hypothermia for neuroprotection. The indications for hypothermia were admitted from the international guidelines and previous research [2].

After each delivery with an asphyxiated newborn, the consecutive term singleton, matched by the delivery hospital, fetal gender, presentation (occipital vs. breech), and the mode of delivery (vaginal, assisted vaginal, elective, emergency, and crash cesarean delivery), was selected as a control. An emergency cesarean was defined as a decision-to-delivery-interval of 30 min and a crash cesarean as an immediate delivery after the decision to deliver. Subgroups were formed based on the mode and onset (spontaneous vaginal delivery, induced vaginal delivery, failed induction and cesarean, cesarean) of delivery.

The data for the study was collected from the hospital database (Siemens Obstetrix). All available information concerning fetal and maternal well-being during pregnancy and delivery was collected. This included maternal age and health (pregestational body mass index (BMI), chronic illnesses, medication), gestation at delivery, parity and previous births, and information concerning hospital visits during the ongoing pregnancy. Data on the time of hospital admission and the time of birth in relation to midwife work shifts was also obtained. We considered and tested multiple previously suggested risk factors for HIE or birth asphyxia [11–24] and analyzed their possible interactions.

### Statistics/analyses

SPSS version 25.0.0 (IBM SPSS Statistics, Armonk, New York) was used to analyze the data. Independent samples t-test and Chi-square test were used for comparing continuous and categorical variables within subgroups, and for testing the independence of variables. Interactions between variables were further assessed with a stratified analysis. (S10)

Crude and adjusted odds ratios (OR and aOR respectively) were calculated using logistic regression to estimate associations between different independent variables and the outcome. From the univariate logistic regression, variables with a *p* < 0.1 were exported to the multivariable logistic regression analysis. A forward stepwise logistic regression analysis was used to suggest multivariable models. Statistical significance was declared at *p* < 0.05 and the CIs were set to 95%. Due to the novel study design and small sample sizes, we saw fit to try out and present two different approaches to the multivariate logistic regression analysis.

We used Benjamini-Hochberg corrected *p*-values (q-values) in the univariate logistic regression to account for multiple testing [25]. Missing values of > 5% per category were imputed using the fully conditional specification method and with maximum iterations of 10.

## Results

During the study period, 98 355 deliveries took place in Helsinki University area hospitals, the average being 19 671 per year. One hundred and twelve term neonates (including one case of 36 + 6 gestational weeks) presented with moderate to severe HIE and were admitted to the NICU to receive therapeutic hypothermia. The study period incidence of therapeutic hypothermia for signs of moderate to severe HIE in our obstetric population was 1.0/1000.

After excluding twin pregnancies, ninety-seven singleton pregnancies with neonatal HIE and therapeutic hypothermia made up the primary study group. Due to the failure to find matched controls, nine more pregnancies were excluded, leaving us with the final study group of eighty-eight cases.

**Fig. 1:**
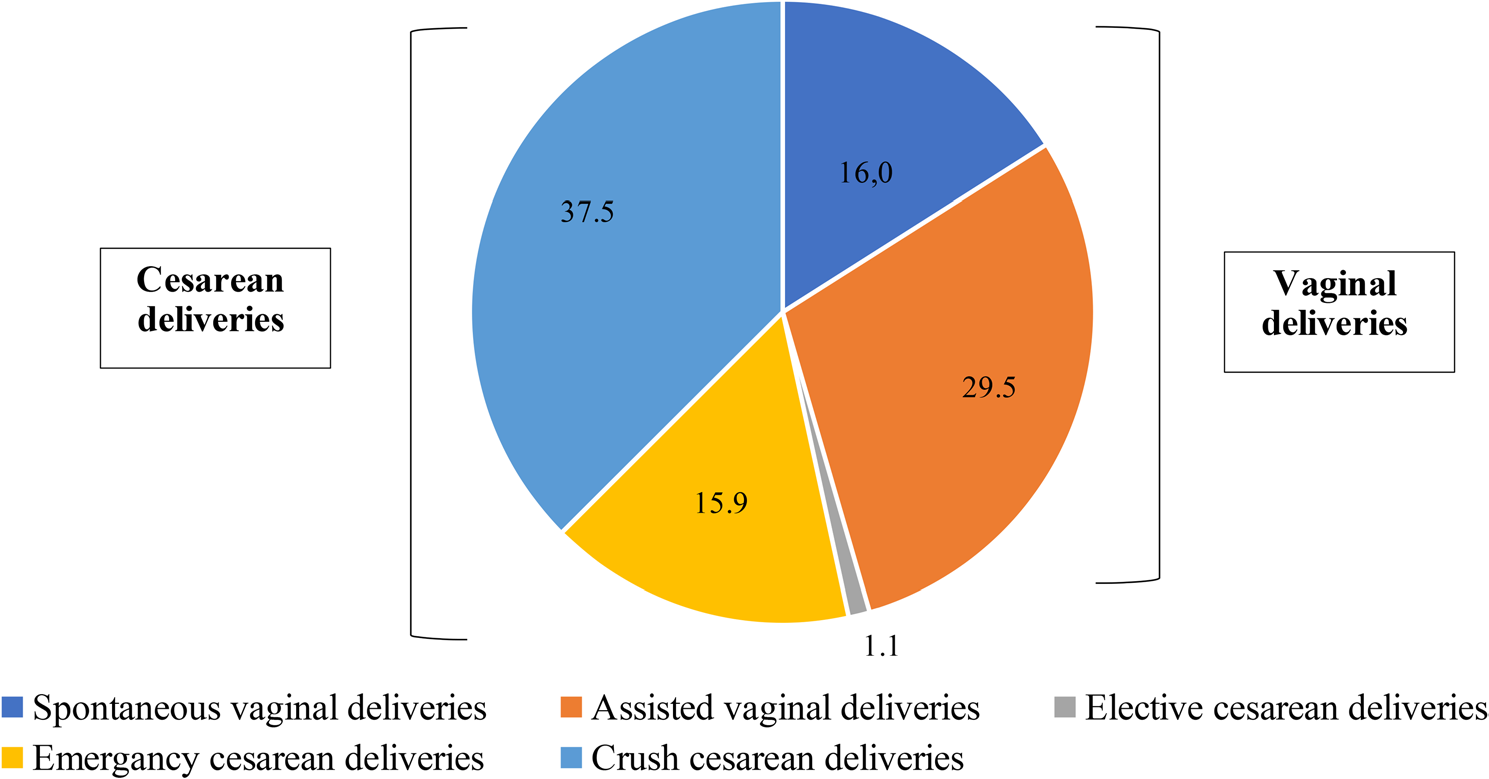
The mode of delivery (%) among cases of hypoxic ischemic encephalopathy and therapeutic hypothermia

Altogether, 45.5% of the neonates (40/88) in the study group were born vaginally and 65% (26/40) of them were ventouse-assisted. Two neonates were vaginally born in breech position. Cesarean deliveries constituted 54.5% (48/88) of study group deliveries, including elective, emergency, and crash procedures with the proportion of 2.0% (1/48), 29.2% (14/48), and 68.8% (33/48) respectively. Four emergency cesarean sections were preceded by a failed instrumental delivery. Due to the matching by delivery mode, the proportions were equal in the control group (Fig. 1: The mode of delivery among cases of HIE and therapeutic hypothermia). The mortality in the study group was 10.2% (9/88). There were no neonatal deaths in the control group.

Approximately the same proportion of patients in the groups were nulliparous (62.5% vs. 59.1%, *p* = 0.576), and no difference was observed in mean maternal age (31.99 vs. 32.96, *p* = 0.239) and BMI (24.20 vs. 23.96, *p* = 0.467). The mean number of daily cigarettes (1.9 vs. 0.4) was higher (*p* = 0.001) in the study group. No difference was detected in the mean gestational age at delivery (38.89 vs. 40.21 gestational weeks, *p* = 0.160) and newborn weight (3458.61 vs. 3472.33 g, *p* = 0.898). Post term pregnancy was more common (3.41% vs. 13.64%, *p* = 0.024) in the control group (Table 1).

**Table 1:** Maternal and infant characteristics in the study groups

	Study group	Control group	OR (95% CI)	*p*-value	q-value	aOR1** (95%CI)	*p*-value	aOR2*** (95% CI)	*p*-value
**Maternal characteristics**									
Nulliparity	55/88 (62.50)	52/88 (59.09)	1.19 (0.65–2.19)	0.576	0.814				
Maternal age	31.99 (5.68)	32.96 (5.19)	0.97 (0.92–1.02)	0.239	0.460				
Maternal BMI*	24.20 (4.52)	23.96 (6.32)	1.02 (0.96–1.08)	0.467	0.778				
Smoking (cigarettes per day, mean) *	1.9 (2.00)	0.4 (1.40)	1.49 (1.16–1.76)	0.001	0.013	1.46 (1.14–1.64)	0.003	1.45 (1.13–1.87)	0.004
**Infant Characteristics**									
Gestational age	39.89 (1.69)	40.21 (1.38)	0.87 (0.71–1.06)	0.160	0.333				
Birth weight	3458.61 (526.62)	3472.33 (506.67)	1	0.898	0.935				
Post term gestation (≥ 42 GW)	3/88 (3.41)	12/88 (13.64)	0.22 (0.06–0.82)	0.024	0.156	0.16 (0.03–1.00)	0.049		

Values are presented as number (%) or mean (SD). BMI=body mass index, GW = gestational weeks. * Missing data (BMI 14,8%, smoking 26.1%) were imputed using the fully conditional specification method and with maximum iterations of 10. Missing data < 5% in other variables. For each model, sensitivity analyses were performed by comparing the pseudo-R-squared values and Akaike Information Criterion (AIC). After testing several models, one model best representing each of the two methods were selected. ** aOR1= adjusted odds ratios acquired from multivariate logistic regression analysis (the first method). All variables adjusted by confounding factors (maternal age, maternal BMI, gestational age, parity, and birth weight). Some confounding factors were used regardless of high *p*-value in the univariate regression analysis. *** aOR2= adjusted odds ratios acquired from multivariate logistic regression analysis (the second method). No assumption of confounding factors. The factors were entered together into the same analysis

There was no difference in the incidence of the most common antenatal complications, such as hypertension or preeclampsia (10.23% vs. 12.50%, *p* = 0.797), intrauterine growth restriction (5.68% vs. 7.95%, *p* = 0.552), gestational (13.64% vs. 15.91, *p* = 0.671) diabetes, type I diabetes (4.55% vs. 3.41%, *p* = 0.701) or suspected chorionamniotis (7.95% vs. 6.82%, *p* = 0.773). There were no cases of diabetes type II in the study group (0% vs. 2.27%, *p* = 0.497) and cholestasis of pregnancy in the control group (3.41% vs. 0%, *p* = 0.246) (Table 2).

**Table 2:** Ante- and intrapartum risk factors in the study and control group

	Study group	Control group	OR (95% CI)	*p*-value	q-value
**Antepartum risk factors**					
Hypertension/preeclampsia	9/88 (10.23)	11/88 (12.50)	0.64 (0.31–2.03)	0.797	0.866
Intrauterine growth restriction	5/88 (5.68)	7/88 (7.95)	0.70 (0.21–2.29)	0.552	0.814
Gestational diabetes mellitus	12/88 (13.64)	14/88 (15.91)	0.84 (0.36–1.92)	0.671	0.866
Diabetes mellitus type I	4/88 (4.55)	3/88 (3.41)	1.35 (0.29–6.21)	0.701	0.866
Diabetes mellitus type II	0/88	2/88 (2.27)	n/a	0.497	n/a
Suspected chorioamnionitis	7/88 (7.95)	6/88 (6.82)	1.18 (0.38–3.67)	0.773	0.866
Cholestasis of pregnancy	3/88 (3.41)	0/88	n/a	0.246	n/a
**Delivery**					
Induction (any method)	19/88 (21.59)	8/88 (9.09)	2.75 (1.13–6.68)	0.025	0.156
Failed induction and cesarean	4/88 (4.55)	10/88 (11.36)	0.37 (0.11–1.23)	0.106	0.265
Phase II duration (min)	35.44 (28.26)	46.38 (32.13)	0.99 (0.97–1.00)	0.098	0.265
Any obstetric emergency	18/88 (20.45)	9/88 (10.23)	2.57 (1.05–6.28)	0.038	0.182
Shoulder dystocia	6/88 (6.82)	0/88	n/a	0.029	n/a
Placental abruption	7/88 (7.95)	4/88 (4.55)	1.82 (0.51–6.44)	0.356	0.636
Uterine rupture	5/88 (5.68)	5/88 (5.68)	1.00 (0.28–3.58)	1	1
**Anesthesia and medication**					
Epidural block	50/88 (56.82)	60/88 (68.18)	0.61 (0.33–1.14)	0.121	0.275
Spinal block	28/88 (31.82)	26/88 (29.55)	1.11 (0.69–2.11)	0.744	0.866
Oral opioid	18/88 (20.45)	21/88 (23.86)	0.82 (0.40–1.67)	0.586	0.814
Oxytocin augmentation	24/88 (27.27)	51/88 (57.95)	0.27 (0.15–0.51)	< 0.001	< 0.001
Nitrous oxide	34/88 (38.64)	47/88 (53.41)	0.55 (0.30–1.00)	0.050	0.182
**Non-medical risk factors**					
Midwife Shift change	40/88 (45.45)	53/88 (60.23)	0.55 (0.30–1.00)	0.051	0.182
Delivery during nightshift (22 − 08)	43/88 (48.86)	33/88 (37.50)	1.74 (0.96–3.18)	0.070	0.219

The values are presented as number (%) or mean (SD)

We detected a higher incidence of labour induction in the study group (21.59% vs. 9.09%, *p* = 0.025), but no difference was detected in the incidence of cesarean after a failed induction (4.55% vs. 11.36%, *p* = 0.106) or the phase II duration of delivery (35.44 min vs. 46.38 min, *p* = 0.098). The overall incidence of any obstetric emergency, i.e., shoulder dystocia, placental abruption, or uterine rupture, was higher (*p* = 0.038) in the study group (20.45% vs. 10.23%), driven by a markedly higher incidence of shoulder dystocia (6.82% vs. 0%, *p* = 0.029).

There was no difference in the use of epidural (56.82% vs. 68.18%, *p* = 0.121), spinal (31.82% vs. 29.55%, *p* = 0.744) or oral opioid (20.45% vs. 23.86%, *p* = 0.586) anesthesia of deliveries, whereas the use of oxytocin augmentation (27.27% vs. 57,95%, *p* < 0.001) and nitrous oxide (38.64% vs. 53.41%, *p* = 0.050) was more common in the control group.

Midwife shift change during the active phase of delivery (45.45% vs. 60.23%, *p* = 0.051) was somewhat more frequent in the control group and the incidence of delivery during the night shift insignificantly more common in the study group (48.86% vs. 37.50%, *p* = 0.070) (Table 2).

The univariate analysis showed that nine independent variables were associated (*p* < 0.1) with either the presence or absence of moderate to severe HIE: Smoking, post term pregnancy, induction of delivery, duration of phase II, any obstetric emergency, augmentation of delivery by oxytocin (all stages of labour, including induction), use of nitrous oxide, shift change of midwives during active delivery, and delivery during night shift (10 pm. to 8 am.).

In the multivariate regression model with four to eight variables in the same model, obstetric emergencies, labour induction and smoking significantly increased the odds of HIE (Table 3, Supporting information Tables S1-S9). We were able to repeat these results in most of the tried models. Induction of labour had a significant association with HIE (*p* = 0.02) in all tried models, but there was no significant association with HIE and the subgroups of induction methods (balloon catheter, vaginal misoprostol, amniotomy followed by oxytocin-infusion), when entered separately to the regression analysis. In fact, in just 33% of cases only one induction method was used.

**Table 3:** The adjusted odds ratios of independent variables associated with hypoxic-ischemic encephalopathy (*p*<0.1) in the univariate analysis

	Study group	Control group	OR (95% CI)	*p*-value	q-value	aOR1** (95%CI)	*p*-value	aOR2*** (95% CI)	*p*-value
**Maternal characteristics**									
Smoking (cigarettes per day) *	1.9 (2.00)	0.4 (1.40)	1.49 (1.16–1.76)	0.001	0.013	1.46 (1.14–1.64)	0.003	1.45 (1.13–1.87)	0.004
**Infant Characteristics**									
Post-term gestation **(**≥ 42 GW)	3 (3.41)	12 (13.64)	0.22 (0.06–0.82)	0.024	0.156	0.16 (0.03–1.00)	0.049		
**Delivery**									
Induction (any method)	19 (21.59)	8 (9.09)	2.75 (1.13–6.68)	0.025	0.156	3.08 (1.18–8.05)	0.02		
Phase II duration	35.44 (28.26)	46.38 (32.13)	0.99 (0.97–1.00)	0.098	0.265	0.99 (0.97-1)	0.12		
Any obstetric emergency	18 (20.45)	9 (10.23)	2.57 (1.05–6.28)	0.038	0.182	2.59 (0.94–7.15)	0.07	3.51 (1.28–9.60)	0.015
**Medication**									
Oxytocin augmentation	24 (27.27)	51 (57.95)	0.27 (0.15–0.51)	< 0.001	< 0.001	0.25 (0.12–0.52)	< 0.001	0.26 (0.13–0.53)	< 0.001
Nitrous oxide	34 (38.64)	47 (53.41)	0.55 (0.30–1.00)	0.050	0.182	0.55 (0.27–1.11)	0.09		
**Non-medical risk factors**									
Midwife Shift change	40 (45.45)	53 (60.23)	0.55 (0.30–1.00)	0.051	0.182	0.70 (0.35–1.41)	0.32		
Delivery during nightshift (22 − 08)	43 (48.86)	33 (37.50)	1.74 (0.96–3.18)	0.070	0.219	1.73 (0.88–3.39)	0.11	1.68 (0.86–3.43)	0.13

Values are presented as number (%) or mean (SD). GW = gestational weeks. * Missing data: smoking 26.1%. Less than 5% in other variables. ** aOR1= adjusted odds ratios acquired from Multivariate logistic regression analysis (the first method). All variables were adjusted by confounding factors (Maternal age, maternal BMI, autoimmune diseases, gestational age, parity, and birth weight). Some confounding factors were used regardless of their high *p*-value in the univariate regression analysis. *** aOR2= adjusted odds ratios acquired from Multivariate logistic regression analysis (the second method). No assumption of confounding factors. The factors were entered together into the same analysis. For each model, sensitivity analyses were performed by comparing the pseudo-R-squared values and Akaike Information Criterion (AIC). After testing several models, one model best representing each of the two methods was selected

In the stratified analysis, the association of induction of labour with HIE was even stronger when oxytocin augmentation was used, OR 9.2 (2.71–31.21). Also, the midwife shift change in induced labours resulted in higher OR for HIE (4.5, 1.73–12.20) (Supporting information, Table S10). When adjusted with other variables in logistic regression, the significant association of oxytocin use and HIE was still strong, while shift change, duration of the second phase of delivery, and delivery during night shift lost their statistical significance (Table 3).

To reveal any common features in different modes of delivery, results were further analyzed in four subgroups: spontaneous and assisted vaginal delivery, and emergency and crash cesarean (Supporting information, Table S11). Mothers without preceding active labour or medical intervention, were omitted from the crash cesarean subgroup.

We also made efforts to deeper analyze the cases of shoulder dystocia and induced labours.

There were six cases of shoulder dystocia in the study group, but none in the control group, which made the regression analysis inapplicable for this specific variable. However, the analyses of all obstetric emergencies (placental abruption, uterine rupture, shoulder dystocia) as a surrogate variable showed a statistically significant association with obstetric emergencies and HIE. The increase in odds of HIE with placental abruption and uterine rupture was insignificant or nonexistent. Aforementioned obstetric emergencies altogether presented an OR of 2.57 and aOR of 3.51 (*p* < 0.05) (Table 3). Other obstetric emergencies, such as cord prolapse and eclampsia, were not present in our data. The analysis of induced labours showed that even though newborns in the study group were heavier (3790 g vs. 3314 g, *p* = 0.030), they were more often born vaginally (84.2% vs. 37.5%, *p* = 0.027) (Supporting information, Table S12).

When analyzed by the mode of delivery, induction was more common in the study group in vaginal (OR 2.75, 95% 1.13–6.68, *p* = 0.016) and assisted (ventouse) vaginal deliveries (*p* = 0.017) (Supporting information, Table S11). The midwife shift change was more common in the control groups of the emergency (*p* = 0.008) and crash (*p* = 0.044) caesarean sections and smoking was more common (*p* = 0.039) in the study group of the crash cesarean subgroup. Five of the six study group cases with shoulder dystocia occurred in the ventouse delivery subgroup (*p* = 0.051) (Supporting information Table S11).

## Discussion

In this study, maternal smoking, induction of labour and obstetric emergencies appeared to be independent risk factors for HIE. There was a clear dose-dependent association with maternal smoking and HIE. This finding prevailed in the multivariate analysis, although the increase in odds remained quite small. There were more induced labours in the study group and the association with labour induction was most pronounced in the subgroup that received oxytocin, accounting for the use during and after induction. Also shoulder dystocia, a poorly predictable obstetric emergency, increased the risk for HIE. Other previously stated antecedents, e.g., nulliparity, gestational age, maternal weight [22], prematurity [15] and chorionamnionitis [6] appeared mostly not to associate with HIE in this study. Furthermore, post term pregnancy, nitrous oxide, and the use of oxytocin as an independent variable had a seemingly opposite association with HIE.

Smoking is known to be a major risk factor for birth asphyxia and HIE. It is strongly associated with antecedents for asphyxia, i.e., fetal growth restriction [26] and the risk of placental abruption [6, 27, 28]. Smoking increases oxidative stress and reduces endogenous defenses in the fetus, which may play a role in the pathogenesis [29]. Even though the harmful effect of smoking is quite indisputable, some bias in the results has to be recognized. The proportion of missing data was substantial, and the imputed data may have skewed the results towards HIE. Also, the frequency, cessation and continuity of smoking was self-reported and susceptible to social desirability bias. It may be, however, safe to assume that the effect of smoking is at least what is presented by the unimputed data (OR 1.21, 95% CI 0.99–1.46, *p* = 0.06).

The association between labour induction and HIE requires careful analysis. Significant multicollinearity between induction of labour and other supposed risk factors (obstetric emergency, oxytocin augmentation, shift change, nitrous oxide, and gestational diabetes) was noticed (Supporting information, Table S13). The induced labours in the study group ended more frequently in vaginal delivery than in the control group. There were no differences in the indications of labour induction. When these factors are weighed in, the independence of induction of labour as a risk factor for HIE can be considered a complex issue.

The role of induction is, however, worth serious consideration, since these pregnancies may include mothers or fetuses with multiple risk factors. In Finland the rate of induced labours has increased from 17.5% in 2007 to 33.9% in 2021 [30]. In addition, the proportion of elective inductions without a medical indication are also increasing [31]. In this study, the risk for HIE was most pronounced among patients with induction of labour together with the use of oxytocin during labour. The oxytocin associated increase in the incidence of encephalopathy was also described in the recent review and meta-analysis by Burgod et al. [32]. It is also worth noticing that even though newborns in the study group of induced labours were heavier, they were more often born vaginally and the number of ventouse deliveries was twice the proportion in the control group. Compared to zero cases in the study group, in approximately one third of control group cases, a crash cesarean followed a failed ventouse delivery. It can be speculated whether some anchoring bias in decision making is involved and the higher proportions of ventouse and vaginal deliveries in the study group and crash cesareans following ventouse trials in the control group reflect the clinicians’ decisions that are associated with the outcome of the newborn. The number of cases is however too small to draw conclusions.

Shoulder dystocia is an obstetric emergency, that results in prolongation of head-to-body delivery, traction of the brachial plexus, and possible birth trauma [33, 34]. The shoulder dystocia incidence reported in studies is approximately 0.7% [35]. Fetal macrosomia is known to increase the risk of shoulder dystocia more than tenfold [35] and in these situations, a planned delivery at early term has been demonstrated to reduce the risk of shoulder dystocia [34]. In this study, six cases of shoulder dystocia were detected in the study group (6.8%) compared to none in the control group. This made the regression analysis inapplicable for this variable. Even though the analysis of all obstetric emergencies (placental abruption, uterine rupture, shoulder dystocia) as a surrogate variable was associated with HIE, the association of HIE with placental abruption and uterine rupture alone was less clear.

As stated, the use of oxytocin in general (irrespective of induction) and nitrous oxide was significantly more common in the control group. However, as shown in the stratified analysis (supplementary information Table S10), in the subgroup of induced labours, oxytocin use was more common in the study group. As the need for induction of labour itself may indicate increased risks in the pregnancy, these variables together increase the risk for adverse outcome. In contrast, spontaneous deliveries with oxytocin augmentation were more frequent in the control group. We suggest that the seemingly protective association of oxytocin augmentation in relation to HIE in the regression models could be explained by the asymmetric distribution of these different subgroups. The same can be speculated for the negative association of the administration of nitrous oxide.

The higher incidence of post-term pregnancies in the control group also needs additional attention. It can be speculated that the need for interventions in control group pregnancies was lower and post term was reached more often. It is also of note, that there was significant collinearity between post term pregnancy and oxytocin administration, midwife shift change and delivery during night shift.

In our study population, 54.5% of patients had a cesarean section and the incidences of emergency and crash cesarean were 15.9% and 37.5%. This describes the underlying existence of ante- and intrapartum complications in the study cohort, since the overall incidences of cesarean sections in the Finnish population were 16.7%, 9.2% and 0.9%, respectively [30]. For example, the rates of pre-eclampsia and pregnancy-induced hypertension in the study were 10.2% and 12.5% compared to our national and worldwide incidences of 5% and 7% [36].

The purpose of this study was to find HIE risk factors that could be anticipated and avoided in the antenatal care and treatment of delivery. For some patients in the study group, the active labour surveillance, and early obstetric interventions, were never at hand. Our efforts in prevention of HIE should be targeted to patients, that during labour are under constant care and observation.

Compared to previous studies, the selection criteria for this study group were different. Although a similar approach with therapeutic hypothermia as surrogate outcome for severe birth asphyxia (and sequential HIE) has been used before [19], most case-control studies rest on a study group of neonates diagnosed with neonatal asphyxia, or with signs of birth asphyxia (low Apgar score and/or signs of acidemia in the peripartum blood samples) [16, 18, 20, 21, 24, 27, 37]. In this study, we chose to use the application of therapeutic hypothermia as the study group inclusion criteria, since it is a clearly defined clinical intervention and in our clinic the indications for use are standardized. The incidence of our inclusion criteria, therapeutic hypothermia (1.1/1000), is slightly higher than the incidence of moderate and severe HIE (0.67/1000) in the study by Liljeström et al. [22]. Although moderate and severe HIE are the main indications for therapeutic hypothermia, the direct comparison of these incidences should be done with caution. Since the exact severity of HIE may still be uncertain immediately after birth (which may have occurred in another hospital) and the decision concerning this undeniably beneficial treatment has to be made within six hours, the incidence of therapeutic hypothermia treatment may be somewhat higher than the exact incidence of diagnosed moderate and severe HIE.

The study setting could be considered a strength of this study. To the best of our knowledge, this was the first case-control study pairing the groups by the mode of delivery, sex, hospital, and fetal presentation at birth. This could partially explain the differences in our results compared to previous similar studies.

The limitations of this study were its retrospective nature and small sample sizes. It is also likely that the matched case control setting together with a small number of cases failed to show the risks associated with previously described risk factors like hypertension, diabetes, and intrauterine growth restriction. These limitations, as well as coincidence, may also explain the higher incidence of post term pregnancies in the control group. The multicollinearity of some studied risk factors also set limitations when interpreting the data.

There were also some restrictions regarding obtaining data. We didn’t have access to primary health care and antenatal outpatient data, and we relied on the history information of the maternity card and database information upon mothers’ admission to the hospital. Chronic illnesses, obstetric complications and infections were not always structurally recorded. Some information such as substance abuse may be underrepresented but unlikely affects our results.

Demographic risk factors, such as social and marital status, are not collected and had to be excluded. Some previously identified risk factors (urinary tract and viral infections) [17, 23, 38, 39] had to be excluded because they are treated at the primary health care level.

After controlling for multiple testing, only two of the univariate logistic regression results (maternal smoking and use of oxytocin) remained statistically significant. When studying rare outcomes in limited sample size, one must be careful not to reject the null hypothesis too readily, while minding possibly important findings that fail to reach nominal statistical significance. We considered both these pitfalls and considered clinical applicability as best we could while interpreting these results, but conclusions based on the findings should still be done with caution.

## Conclusion

According to our results, induction of labor may be an independent risk factor for HIE, and it should only be used in situations where it evidently improves the outcome of labour. Special vigilance is required from the obstetric team when deciding upon induction and when managing these patients during labour. The increased risk of HIE associated with smoking and obstetric emergencies is unfortunately mostly out of the clinician’s reach.

### Electronic supplementary material

Below is the link to the electronic supplementary material.

Supplementary Material 1

## Data Availability

The data that support the findings of this study are available from the corresponding author upon reasonable request.

## Abbreviations

HIE: Hypoxic-ischemic encephalopathy
NICU: Neonatal Intensive Care Unit
OR: Odds ratio
