# Supplementary material for: Risk factors for neonatal hypoxic ischemic encephalopathy and therapeutic hypothermia: a matched case-control study
Source: BMC Pregnancy Childbirth. 2024 Jun 12;24:421. doi: 10.1186/s12884-024-06596-8 (PMC11167761; doi:10.1186/s12884-024-06596-8)
Supplement: Supplementary file 1 — Supplementary Material 1 [file 12884_2024_6596_MOESM1_ESM.docx]

**Supporting information**

1. **Regression models**
   1. Models 1 and 2: Variables with a *p*-value < 0.1 in univariate regression analysis were adjusted one by one with possible confounding factors.
   2. Models 3-8: Only variables with a *p*-value <0.1 in univariate regression analysis.

The following references were used to choose the tested confounded variables:

1. Kappel B, Eriksen G, Hansen KB, Hvidman L, Krag-Olsen B, Nielsen J, et al. Short Stature in Scandinavian Women: An obstetrical risk factor. Acta Obstet Gynecol Scand [Internet]. 1987 Jan 1 [cited 2020 Jun 18];66(2):153–8. Available from: http://doi.wiley.com/10.3109/00016348709083038

2. Kurinczuk JJ, White-Koning M, Badawi N. Epidemiology of neonatal encephalopathy and hypoxic-ischaemic encephalopathy. Vol. 86, Early Human Development. 2010. p. 329–38.

3. Lundgren C, Brudin L, Wanby AS, Blomberg M. Ante- and intrapartum risk factors for neonatal hypoxic ischemic encephalopathy. J Matern Neonatal Med. 2018 Jun 18;31(12):1595–601.

4. Nelson DB, Lucke AM, Mcintire DD, Sánchez PJ, Leveno KJ, Chalak LF. Obstetric Antecedents to Body Cooling Treatment of the Newborn Infant. Am J Obs Gynecol. 2014;211(2):155–6.

5. Badawi N, Kurinczuk JJ, Keogh JM, Alessandri LM, O’Sullivan F, Burton PR, et al. Antepartum risk factors for newborn encephalopathy: The Western Australian case-control study. Br Med J. 1998 Dec 5;317(7172):1549–53.

6. Ellis M, Manandhar N, Manandhar DS, De L Costello AM. Risk factors for neonatal encephalopathy in Kathmandu, Nepal, a developing country: Unmatched case-control study. Br Med J. 2000 May 6;320(7244):1229–36.

7. Locatelli A, Incerti M, Paterlini G, Doria V, Consonni S, Provero C, et al. Antepartum and intrapartum risk factors for neonatal encephalopathy at term. Am J Perinatol [Internet]. 2010 Sep [cited 2020 Mar 2];27(8):649–54. Available from: http://www.ncbi.nlm.nih.gov/pubmed/20225171

1. **Stratified analysis and subgroup analysis**
   1. Stratified analysis. Testing the independence of the variables before forming multivariable logistic regression models (Table 10).
   2. The subgroup analysis of risk factors by the mode of delivery (Table 11)
   3. The subgroup analysis of successful labour inductions based on the indication of induction (Table 12).

The following references were used to choose the tested variables:

1. Nelson DB, Lucke AM, Mcintire DD, Sánchez PJ, Leveno KJ, Chalak LF. Obstetric Antecedents to Body Cooling Treatment of the Newborn Infant. Am J Obs Gynecol. 2014;211(2):155–6.

2. Lindsey JK, Jones B. Choosing among generalized linear models applied to medical data. Vol. 17, Statistics in Medicine. Stat Med; 1998. p. 59–68.

3. Liljestrom L, Wikstrom AK, Agren J, Jonsson M. Antepartum risk factors for moderate to severe neonatal hypoxic ischemic encephalopathy: a Swedish national cohort study. Acta Obstet Gynecol Scand. 2018 May 1;97(5):615–23.

4. Ellis M, Manandhar N, Manandhar DS, De L Costello AM. Risk factors for neonatal encephalopathy in Kathmandu, Nepal, a developing country: Unmatched case-control study. Br Med J. 2000 May 6;320(7244):1229–36.

5. Lundgren C, Brudin L, Wanby AS, Blomberg M. Ante- and intrapartum risk factors for neonatal hypoxic ischemic encephalopathy. J Matern Neonatal Med. 2018 Jun 18;31(12):1595–601.

6. Badawi N, Kurinczuk JJ, Keogh JM, Alessandri LM, O’Sullivan F, Burton PR, et al. Antepartum risk factors for newborn encephalopathy: The Western Australian case-control study. Br Med J. 1998 Dec 5;317(7172):1549–53.

7. Kappel B, Eriksen G, Hansen KB, Hvidman L, Krag-Olsen B, Nielsen J, et al. Short Stature in Scandinavian Women: An obstetrical risk factor. Acta Obstet Gynecol Scand. 1987 Jan 1;66(2):153–8.

8. Kurinczuk JJ, White-Koning M, Badawi N. Epidemiology of neonatal encephalopathy and hypoxic-ischaemic encephalopathy. Vol. 86, Early Human Development. 2010. p. 329–38.

**Tables:**

Table S1. Regression model 1

Table S2. Regression model 2

Table S3. Regression model 3

Table S4. Regression model 4

Table S5. Regression model 5

Table S6. Regression model 6

Table S7. Regression model 7

Table S8. Regression model 8

Table S9. The comparison of AIC-values from the different models

Table S10. Stratified analysis. Testing the independence of the variables before forming multivariable logistic regression models

Table S11. The subgroup analysis of risk factors by the mode of delivery

Table S12. A regression analysis of pregnancies with a successful induction

Table S13. The collinearity of independent variables

**Table S1.** Regression model 1

| **Independent variable** | **OR** | ***p-*value** | **CI (95 %)** | **AIC** |
| --- | --- | --- | --- | --- |
| Night shift | 1.59 | 0.17 | 0.82-3.06 | 230 |
| Midwife shift change | 0.66 | 0.25 | 0.33-1.34 | 230 |
| Post term pregnancy | 0.14 | 0.03* | 0.03-0.80 | 220 |
| Phase II duration | 0.98 | 0.07 | 0.96-1.00 | 115 |
| Obstetric emergency | 2.89 | 0.04* | 1.06-7.88 | 230 |
| Oxytocin augmentation | 0.26 | <0.001 | 0.12-0.53 | 210 |
| Nitrous oxide | 0.56 | 0.09 | 0.28-1.10 | 230 |
| Smoking | 1.50 | 0.002* | 1.17-1.92 | 230 |

All variables were adjusted by maternal age, body mass index, diabetes mellitus, hypertension/preeclampsia, parity, birth weight and smoking

**Table s2.** Regression model 2

| **Independent variable** | **OR** | ***p-*value** | **CI (95 %)** | **AIC** |
| --- | --- | --- | --- | --- |
| Night shift | 1.73 | 0.11 | 0.88-3.39 | 220 |
| Midwife shift change | 0.70 | 0.32 | 0.35-1.41 | 225 |
| Post term pregnancy | 0.16 | 0.049 | 0.03-1.00 | 220 |
| Phase II duration | 0.99 | 0.12 | 0.97-1 | 120 |
| Obstetric emergency | 2.59 | 0.07 | 0.938-7.15 | 225 |
| Oxytocin augmentation | 0.25 | <0.001 | 0.12-0.52 | 210 |
| Nitrous oxide | 0.55 | 0.09 | 0.27-1.11 | 222 |
| Induction of labour | 3.08 | 0.02 | 1.18-8.05 | 220 |
| Smoking | 1.46 | 0.003 | 1.46-1.14 | 220 |

All variables were adjusted by maternal age, body mass index, autoimmune diseases, gestational age, parity, birth weight, smoking

**Models 3 –**

**Regression models 3-8:** Only variables with a *p*-value <0.1 in one variable regression analysis

**Table S3.** Regression model 3

| **Independent variable** | **OR** | ***p-*value** | **CI (95 %)** |
| --- | --- | --- | --- |
| Induction of labour | 4.91 | 0.008 | 1.51-16.02 |
| Night shift | 1.82 | 0.23 | 0.67-4.80 |
| Post term pregnancy | 0.07 | 0.06 | 0.005-1.07 |
| Phase II duration | 0.18 | 0.99 | 0.97-1.01 |
| Smoking | 1.63 | 0.07 | 0.96-2.75 |

| **Independent variable** | **OR** | ***p-*value** | **CI (95 %)** |
| --- | --- | --- | --- |
| Oxytocin augmentation | 0.27 | <0.001 | 0.13-0.56 |
| Night shift | 2.02 | 0.06 | 0.98-4.14 |
| Post term pregnancy | 0.15 | 0.03 | 0.03-0.86 |
| Obstetric emergency | 3.36 | 0.02 | 1.19-9.43 |
| Smoking | 1.45 | 0.005 | 1.12-1.88 |

All variables in the same regression model

**Table S4.** Regression model 4

All variables in the same regression model

**Table S5.** Regression model 5

| **Independent variable** | **OR** | ***p-*value** | **CI (95 %)** |
| --- | --- | --- | --- |
| Night shift | 1.997 | 0.052 | 9.994 – 4.01 |
| Post term pregnancy | 0.117 | 0.019 | 0.019-0.71 |
| Obstetric emergency | 2.696 | 0.055 | 0.981-7.415 |
| Induction of labour | 3.045 | 0.027 | 1.134-8.177 |
| Smoking | 1.51 | 0.002 | 1.17–1.95 |

All variables in the same regression model

**Table S6.** Regression model 6

All variables in the same regression model

| **Independent variable** | **OR** | ***p-*value** | **CI (95 %)** |
| --- | --- | --- | --- |
| Oxytocin augmentation | 0.27 | <0.001 | 0.14-0.52 |
| Night shift | 2.16 | 0.02 | 1.11-4.23 |
| Post term pregnancy | 0.23 | 0.04 | 0.06-0.95 |
| Obstetric emergency | 3.14 | 0.02 | 1.17-8.40 |

**Table S7.** Regression model 7

| **Independent variable** | **OR** | ***p-*value** | **CI (95 %)** |
| --- | --- | --- | --- |
| Post term pregnancy | 0.17 | 0.04 | 0.032-0.935 |
| Nitrous oxide | 0.65 | 0.49 | 0.33-1-25 |
| Obstetric emergency | 2.65 | 0.048 | 1.01-6.92 |
| Smoking | 1.50 | 0.002 | 1.16-1.93 |

All variables in the same regression model

**Table S8.** Regression model 8

All variables in the same regression model

| **Independent variable** | **OR** | ***p-*value** | **CI (95 %)** |
| --- | --- | --- | --- |
| Post term pregnancy | 0.166 | 0.011 | 0.042-0.662 |
| Induction of labour | 2.863 | 0.029 | 1.112-7.369 |
| Obstetric emergency | 2.401 | 0.071 | 0.929-6.204 |
| Night shift | 2.127 | 0.022 | 1.115-4.056 |

The interpretation of AIC values from different models using the same data: The AIC decreases as the number of independent variables is reduced. On the other hand, the Hosmer-Lemeshow goodness of fit test, gave worse values with fewer variables in one model.

| **Models** | **AIC** |
| --- | --- |
| 1 | 212 |
| 2 | 209 |
| 3 | 110 |
| 4 | 106 |
| 5 | 111 |
| 6 | 45 |
| 7 | 100 |
| 8 | 37 |

**Table S9.** The comparison of AIC-values from the different models

| **Table S10.** Stratified analysis. Testing the independence of the variables before forming multivariable logistic regression models | **Cases** | **Controls** | **OR (95 % CI)** | ***p*-value** |
| --- | --- | --- | --- | --- |
| **Oxytocin augmentation** | **24 (27.27)** | **51 (57.95)** | **0.27 (0.15–0.51)** | **<0.001** |
| Induction of labour + | 12/19 (63.2) | 5/8 (62.5) | 1.03 (0.19–5.68) | 0.97 |
| Induction of labour - | 12/69 (17.4) | 46/80 (57.5) | 0.16 (0.07–0.33) | <0.001 |
| Shift change + | 19/40 (47.5) | 36/53 (67.9) | 0.43 (0.18–1.00) | 0.05 |
| Shift change - | 5/48 (10.4) | 15/35 (42.9) | 0.16 (0.05–0.49) | 0.001 |
| Nitrous oxide + | 14/34 (41.2) | 33/47 (70.2) | 0.30 (0.12–0.75) | 0.009 |
| Nitrous oxide - | 10/54 (18.5) | 18/41 (43.9) | 0.30 (0.12–0.73) | 0.007 |
| Post term pregnancy + | 2/3 (66.7) | 8/12 (66.7) | 1.00 (0.07-14.64) | 1.00 |
| Post term pregnancy - | 22/85 (25.9) | 43/76 (56.6) | 0.27 (0.14-0.52) | <0.001 |
| **Induction of labour** | **19 (21.59)** | **8 (9.09)** | **2.75 (1.13–6.68)** | **0.025** |
| obstetric emergency + | 6/18 (33.3) | 1/9 (11.1) | 4.00 (0.40-39.83) | 0.36 |
| obstetric emergency - | 13/79 (18.6) | 7/79 (8.9) | 2.34 (0.88-6.27) | 0.085 |
| oxytocin augmentation + | 12/24 (50.0) | 5/51 (9,8) | 9.2 (2.71-31.21) | <0.001 |
| oxytocin augmentation - | 7/64 (10.9) | 3/37 (8.1) | 1.39 (0.34-5.74) | 0.74 |
| shift change + | 18/40 (45.0) | 8/53 (15.1) | 4.5 (1.73-12.20) | 0.001 |
| shift change - | 1/48 (2.1) | 0/35 (0) | 0.57 (0.48-0.69) | 1.00 |
| nitrous oxide + | 13/34 (38.2) | 6/47 (12.8) | 4.23 (1.41-12.72) | 0.008 |
| nitrous oxide - | 6/54 (11.1) | 2/41 (4.9) | 2.44 (0.47-12.76) | 0.46 |
| GDM + | 5/12 (41.7) | 4/14 (28.6) | 1.79 (0.35-9.13) | 0.68 |
| GDM - | 14/76 (18.4) | 4/74 (5.4) | 3.95 (1.24-12.64) | 0.02 |
| **Shift change** | **40 (45.45)** | **53 (60.23)** | **0.55 (0.30–1.00)** | **0.051** |
| Induction of labour + | 18/19 (94.7) | 8/8 (100) | 1.44 (1.12–1.87) | 1.00 |
| Induction of labour - | 22/69 (31.9) | 45/80 (56.3) | 0.36 (0.19–0.71) | 0.003 |
| Oxytocin augmentation + | 19/24 (79.2) | 36/51 (70.6) | 1.58 (0.50–5.02) | 0.43 |
| Oxytocin augmentation - | 21/64 (32.8) | 17/37 (45.9) | 0.58 (0.25–1.32) | 0.19 |
| Nitrous oxide + | 26/34 (76.5) | 35/47 (74.5) | 1.11 (0.40–3.12) | 0.84 |
| Nitrous oxide - | 14754 (25.9) | 18/41 (43.9) | 0.45 (0.19–1.06) | 0.66 |
| DM I + | 0/4 (0) | 1/3 (33.3) | 0.33 (0.11–1.03) | 0.43 |
| DM I - | 40/84 (47.6) | 52/85 (61.2) | 0.58 (0.31–1.06) | 0.78 |
| Post term pregnancy + | 2/3 (66.7) | 10/12 (83.3) | 0.40 (0.02–6.85) | 0.52 |
| Post term pregnancy - | 38/85 (44.7) | 43/76 (56.6) | 0.62 (0.33–1.16) | 0.13 |
| **Nitrous oxide** | **34 (38.64)** | **47 (53.41)** | **0.55 (0.30–1.00)** | **0.050** |
| Shift change + | 26/40 (65.0) | 35/53 (66.0) | 0.96 (0.40–2.27) | 0.92 |
| Shift change - | 8/48 (16.7) | 12/35 (34.3) | 0.38 (0.14–1.08) | 0.06 |
| Induction of labour + | 13/19 (68.4) | 6/8 (75.0) | 0.72 (0.11–4.69) | 0.73 |
| Induction of labour - | 21/69 (30.4) | 41/80 (51.2) | 0.42 (0.21–0.82) | 0.01 |
| Oxytocin augmentation + | 14/24 (41.7) | 33/51 (64.7) | 0.76 (0.28–2.06) | 0.60 |
| Oxytocin augmentation - | 20/64 (31.3) | 14/37 (37.8) | 0.75 (0.32–1.75) | 0.50 |
| **Post term pregnancy** | **3 (3.41)** | **12 (13.64)** | **0.22 (0.06–0.82)** | **0.024** |
| Shift change + | 2/40 (5.0) | 10/53 (18.9) | 0.27 (0.45–1.10) | 0.06 |
| Shift change - | 1/48 (2.1) | 2/35 (5.7) | 0.35 (0.03–4.03) | 0.57 |
| Oxytocin augmentation + | 2/24 (8.3) | 8/51 (15.7) | 0.49 (0.96–2.5) | 0.38 |
| Oxytocin augmentation - | 1/64 (1.6) | 4/37 (10.8) | 0.13 (0.14–1.22) | 0.06 |
| Night shift + | 3/43 (7.0) | 7/33 (21.2) | 0.30 (0.07–1.12) | 0.09 |
| Night shift - | 0/45(0) | 5/55 (9.1) | 0.53 (0.44–0.64) | 0.06 |
| **Obstetric emergency** | **18 (20.45)** | **9 (10.23)** | **2.57 (1.05–6.28)** | **0.038** |
| Induction of labour + | 6/19 (31.6) | 1/8 (12.5) | 3.23 (0.32–32.48) | 0.063 |
| Induction of labour - | 12/69 (17.4) | 8/80 (10.0) | 1.90 (0.74–4.95) | 0.19 |
| **Night shift** | **43 (48.86)** | **33 (37.50)** | **1.74 (0.96–3.18)** | **0.070** |
| Post term pregnancy + | 3/3 (100) | 7/12 (58.3) | 1.43 (0.95–2.14) | 0.17 |
| Post term pregnancy - | 40/85 (47.1) | 26/76 (34.2) | 1.71 (0.90–3.23) | 0.98 |

**Table S11**. The subgroup analysis of risk factors by the mode of delivery


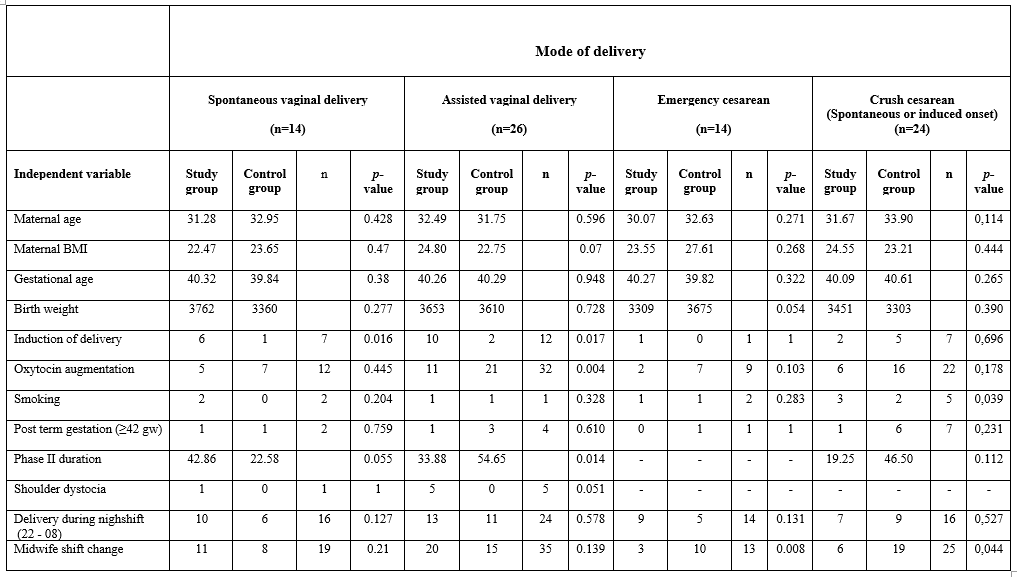


* Cases without a preceding active labour or medical intervention were omitted from the group.

BMI=body mass index, GW=gestational weeks. The comparisons were made by using the independent samples T-test.

The comparisons were made by using the independent samples T-test.



|  | **Study group**  **(n=19)** | **Control group**  **(n=8)** | ***p*-value** |
| --- | --- | --- | --- |
| Maternal age | 32.2 (5.46) | 30.8 (4.28) | 0.526 |
| Maternal body mass index | 24.7 (4.67) | 22.2 (1.75) | 0.094 |
| Nulliparity | 12 (63.16) | 6 (75.0) | 0.676 |
| Gestational age (years) | 39.9 (1.94) | 40.6 (1.59) | 0.367 |
| Birth weight (g) | 3790 (509.6) | 3314 (424.6) | 0.030 |
| Delivery during midwife nightshift (22 - 08) | 10 (52.6) | 3 (37.5) | 0.678 |
| **Indication of induction** |  |  |  |
| Diabetes mellitus + fetal macrosomia | 4 (21.1) | 0 (0) | 0.285 |
| Post-term pregnancy | 6 (31.6) | 2 (25.0) | 1 |
| Hypertension/ preeclampsia | 4 (21.1) | 2 (25.0) | 1 |
| Ruptured membranes | 1 (5.3) | 2 (25.0) | 0.201 |
| Other indications | 4 (21.1) | 2 (25.0) | 1 |
| Delivery duration from induction to birth (hours) | 24.7 (13.48) | 23.9 (19.27) | 0.891 |
| **Mode of delivery** |  |  |  |
| All vaginal deliveries | 16 (84.2) | 3 (37.5) | 0.027 |
| Vaginal (unassisted) | 6 (31.6) | 1 (12.5) | 0.646 |
| Ventouse delivery | 10 (52.6) | 2 (25.0) | 0.677 |
| Emergency cesarean section | 1 (5.3) | 0 (0) | 1 |
| Crash cesarean section | 3 (15.8) | 2 (25.0) | 1 |
| Failed ventouse + crash cesarean section | 0 (0) | 3 (37.5) | 0.06 |
| Shoulder dystocia | 3 (15.8) | 0 (0) | 0.529 |

**Table S12**. A regression analysis of pregnancies with a successful induction



**Table S13**. The collinearity of independent variables

| **Night**  **shift** | ***p-value*** | 0.068 |  |  |  |  |
| --- | --- | --- | --- | --- | --- | --- |
|  | Independent  variable | Post  term pregnancy |  |  |  |  |
| **Obstetric**  **emergency** | ***p-value*** | 0.084 |  |  |  |  |
|  | Independent  variable | Induction  of  labour |  |  |  |  |
| **Post term**  **pregnancy** | ***p-value*** | 0.028 | 0.049 | 0.068 |  |  |
|  | Independent  variable | Midwife  shift  change | Oxytocin  augmentation | Night  shift |  |  |
| **Nitrous**  **oxide** | ***p-value*** | <0.001 | 0.06 | <0.001 |  |  |
|  | Independent  variable | Midwife  shift  change | Induction  of  labour | Oxytocin  augmentation |  |  |
| **Midwife**  **shift**  **change** | ***p-value*** | <0.001 | <0.001 | <0.001 | 0.53 | 0.028 |
|  | Independent  variable | Induction  of  labour | Oxytocin  augmentation | Nitrous  oxide | DM I | Post  GDM=gestational diabetes mellitus, DM I=diabetes mellitus type 1  term pregnancy |
| **Induction**  **of**  **labour** | ***p-value*** | 0.084 | 0.02 | <0.001 | 0.06 | 0.007 |
|  | Independent  variable | Obstetric emergency | Oxytocin  augmentation | Midwife  shift  change | Nitrous  oxide | GDM |
| **Oxytocin**  **augmentation** | ***p-value*** | 0.02 | <0.001 | <0.001 | 0.049 |  |
|  | Independent  variable | Induction  of  labour | Midwife  shift  change | Nitrous  oxide | Post term  pregnancy |  |
